# Frequently Asked Questions about Thrombosis during COVID-19 Pandemic

**DOI:** 10.1055/s-0040-1718884

**Published:** 2020-10-20

**Authors:** Lolita Golemi, Peter Hanson, Alfonso Tafur

**Affiliations:** 1 Rush Medical College, Chicago, Illinois, United States; 2 NorthShore University HealthSystem, Evanston, Illinois, United States; 3 Department of Medicine, Division of Cardiovascular Medicine, NorthShore University HealthSystem, Evanston, Illinois, United States

**Keywords:** COVID-19, deep vein thrombosis, pulmonary embolism, anticoagulants

## Abstract

The coronavirus disease 2019 (COVID-19) pandemic and the “shelter-in-place” orders have placed a significant strain on patients and providers. We believe that patient education is crucial during these times, so together with their health care providers, the patients can make the best decisions in regard to their health. Anchored on a patient-perspective, we summarize frequently asked questions illustrating a growing thrombosis concern.

## Introduction

The coronavirus disease 2019 (COVID-19) pandemic and the “shelter-in-place” orders have placed a significant strain on patients and providers. As we continue to discover new information about this virus, there is ongoing uncertainty in the patients' lives. We believe that patient education is crucial during these times, so together with their health care providers, the patients can make the best decisions in regard to their health. Anchored on a patient-perspective, we summarize frequently asked questions illustrating a growing thrombosis concern.

1. As a patient who has had a deep vein thrombosis (DVT) and pulmonary embolism (PE) several years ago, what specific daily routines should I incorporate into my lifestyle?

The World Health Organization (WHO) recommends that all adults aged 18 to 64 should participate in at least 150 minutes of moderate-intensity aerobic physical activity or at least 75 minutes of vigorous-intensity aerobic physical activity throughout the week. Aerobic activities include walking, running, dancing, hiking amongst other forms of exercise. With the shelter-in-place orders, there is limited access to fitness gyms and public parks, thus, staying physically active has become more challenging. WHO suggests that following online exercise classes, standing up or even walking around the house can be considered adequate physical activity, given the circumstances. 1

Patients who have suffered a venous thromboembolism (VTE), which includes DVT and PE, should follow these guidelines strictly, as they are at a higher risk for getting another blood clot if they remain inactive for long periods of time.

WHO has also put out recommendations for a healthy diet during quarantine. They recommend home cooked meals, fruits, and vegetables over nonperishable goods. They also emphasize the importance of monitoring portion sizes and limiting sugar, salt, and alcohol intake. 2 Although greens are highly recommended, patients on warfarin should keep consistency in vitamin K-rich food consumption, not to reverse the effect of the medication. 3

2. I have read that COVID-19 patients (in Wuhan) that died had traces of DVT and even PE. Did they have them before contracting COVID-19, or did COVID-19 cause the DVT/PE? What are your thoughts on which came first and should blood thinner users increase the dose of their medications to prevent clotting?

Severe acute respiratory syndrome coronavirus 2 (SARS-CoV2) infection has been associated with an increase in formation of blood clots in patients who did not necessarily have one prior to the hospitalization. 4

As of this writing, there is not enough scientific evidence that recommends any benefits in increasing the dose of blood thinners in non-COVID-19 patients. 5 It is crucial not to make any medication changes without consulting your doctor, because increasing the dose of blood thinners can increase the risk of bleeding. For patients on warfarin, this pandemic may have presented some challenges, as these patients require frequent international normalized ratio (INR) checks. It is important to talk to your physician and discuss if you could be switched to a different medication that requires less frequent checks, to avoid having to leave your house during these times. In addition, several institutions have implemented a drive through INR testing for patients who cannot be changed to other medications.

3. Would having a test of my D-dimer on a weekly basis assist in watching out? If not, is there any advantage to a more frequent test of D-dimer during this time?

D-dimer testing, which uses a blood specimen, is not used as a preventative measure to detect COVID-19. The Centers for Disease and Control Prevention (CDC) states the best method to confirm if you have contracted the coronavirus is through an upper respiratory specimen. 6 Some of the symptoms you should be looking for are fever, cough, shortness of breath, and muscles aches. There is currently no evidence that supports getting regular D-dimer levels would be necessary. However, in patients with COVID-19, D-dimer testing can help select patients at higher risk. 7 If you are not experiencing any of the symptoms for a blood clot which can include shortness of breath, swelling of an extremity, tenderness, or warm sensation, a D-dimer level check will most likely not be needed.

4. What other steps does a DVT/PE patient need to do to avoid contracting COVID-19?

Patients who have had a DVT or PE are not at a higher risk than the general public for contracting the coronavirus. The precautions recommended by Center of Disease Control should be the cornerstone for these patients as well. Some of these precautions include:

- Washing your hands often with soap and water for at least 20 seconds.
- If soap and water are not readily available, using hand sanitizer that contains at least 60% alcohol can be sufficient.
- Make sure to wear a cloth face cover when going out in public.
- Clean and disinfect frequently touched surfaces daily, including tablets, doorknobs, light switches countertops, handles, desks, phones, keyboards, toilets, and sinks. 8

Chronically anticoagulated patients need to be cautious when attending anticoagulation clinics.

5. Should we consider getting tested? I have had a dry cough since last September that has not gotten worse or better, but no fever.

Given the length of your symptoms, it is highly unlikely that they are due to COVID-19. CDC has published guidance for who should be tested, but the decisions about testing are made by state and local health departments. If you currently are experiencing new symptoms such as shortness of breath, cough and fevers, muscle aches you should contact your health care provider.

6. If I do contract COVID-19, what is the increase in death estimates than if I did not have a DVT/PE? Will the symptoms be more severe?

Having had a PE or DVT puts you in a group of patients who are at higher risk for developing COVID-19 complications. 9 There is yet a lot of research to be done on this and having an exact number of how much higher the mortality rate is in those who had a prior blood clot has yet to be determined.

## Conclusion

The information on COVID-19 is still evolving. We are learning more about this virus each day, and the guidelines are adjusted based on latest research findings. As of this writing, we still do not have clear guidelines on what specific changes VTE patients can make to their medications to reduce the chances of clotting, as they are forced to spend more time indoors. However, we remain hopeful that as new research comes out, more of our patients' questions will be answered.
